# The rheological properties of different GNPs

**DOI:** 10.1186/1476-511X-11-14

**Published:** 2012-01-24

**Authors:** Mohamed Anwar K Abdelhalim

**Affiliations:** 1 Department of Physics and Astronomy, College of Science, King, Saud University, Saudi Arabia

**Keywords:** GNPs, rheological properties, size, temperature

## Abstract

**Background:**

Rheological analysis can be employed as a sensitive tool in predicting the physical properties of gold nanoparticles (GNPs). Understanding the rheological properties of GNPs can help to develop a better therapeutic cancer product, since these physical properties often link material formulation and processing stages with the ultimate end use. The rheological properties of GNPs have not been previously documented. The present study attempted to characterize the rheological properties of different sizes of GNPs at: 1) fixed temperature and wide range of shear rates; 2) varied temperature and fixed shear rate.

**Methods:**

10, 20 and 50 nm GNPs was used in this study. Several rheological parameters of GNPs such as viscosity, torque%, shear stress and shear rate were evaluated using Brookfield LVDV-III Programmable rheometer supplied with temperature bath and controlled by a computer. To measure fluid properties (viscosity as function of shear rate), e.g., to determine whether the flow is Newtonian or non-Newtonian flow behaviour, and viscoelasticity (viscosity as function of temperature), rheological parameters were firstly measured at starting temperature of 37°C and wide range of shear rates from 375 to 1875 s^-1^, and secondly at a gradual increase of temperature from 37 to 42°C and fixed shear rate of 1875 s^-1^.

**Results:**

The 10, 20 and 50 nm GNPs showed mean size of 9.45 ± 1.33 nm, 20.18 ± 1.80 nm, and 50 nm GNPs, respectively. The 10 and 20 nm GNPs showed spherical morphology while 50 nm GNPs showed hexagonal morphology using the transmission electron microscope (TEM). The relation between viscosity (cp) and shear rate (s^-1^) for 10, 20 and 50 nm GNPs at a temperature of 37°C showed non-Newtonian behaviour. Although the relationship between SS (dyne/cm^2^) and SR (s^-1^) for 10, 20 and 50 nm GNPs was linearly related however their fluid properties showed non-Newtonian behaviour.

**Conclusions:**

The torque%, viscosity **(cp) **and SS (dyne/cm^2^) of all GNP sizes decreased with increasing the temperature and with decreasing the GNP size (for each fixed temperature value). For each shear rate value, the viscosity of all GNPs decreased with decreasing the GNP size. This study demonstrates that the physical, dimensional and morphological changes of GNPs have effective influence on their rheological properties. To understand and categorize the role of GNPs in drug delivery and cancer therapy, GNPs of varying size, number of particles, shape and surface should be taken into consideration. Moreover, further additional in vivo studies after administration of GNPs in rats should be performed to support this hypothesis.

## Background

Nanotechnology is enabling technology that deals with nano-meter sized objects. A study on nanoparticle is becoming a hot point owing to their novel physical and chemical attributes in electronics and optics [1-3]. The GNPs have unique optical properties such as distinctive extinction bands in the visible region, due to surface plasmon oscillation of the free electrons [4].

Nanoparticles (NPs) may differ in reactivity and solubility and may interact with all kinds of endogenous proteins, lipids, polysaccharides, and cells. Based on experiences in inhalation toxicology, a series of tests was proposed for evaluating the toxicity of NPs used in the drug delivery systems [5].

Gold in its bulk form has been considered an inert, noble metal with some therapeutic and medicinal value. GNPs of smaller sizes are thought to be relatively non-cytotoxic [6]. The particle size-dependent organ distribution of GNPs has been studied in vivo [7-9].

In a Newtonian fluid, the relation between the shear stress and the shear rate is linear, passing through the origin, the constant of proportionality being the coefficient of viscosity. A Newtonian fluid that has a constant viscosity at all shear rates (at a constant temperature and pressure), and can be described by a one-parameter rheological model. In a non-Newtonian fluid, the relation between the shear stress and the shear rate is different, and can even be time-dependent. Therefore a constant coefficient of viscosity cannot be defined [10].

The measurement of rheological properties is applicable to all material types, from fluids to semi-solids and even solids such as polymers and composites [11-14]. Rheology is defined as the flow of fluids and deformation of solids under applied stresses or strains.

Whole-blood viscosity is a predictor of stroke, carotid intima-media thickening, and carotid atherosclerosis. However, in most studies, whole blood viscosity was measured at a few non-specific shear rates, and these data do not reflect the complete rheological characteristics found in these studies [15-17].

Erythrocyte deformability refers to the cellular properties of erythrocytes (red blood cells) that determine the degree of shape change under a given level of applied force. Erythrocytes change their shape extensively under the influence of applied forces in fluid flow or while passing through microcirculation. The extent and geometry of this shape change is determined by the mechanical properties of erythrocytes, the magnitudes of the applied forces and the orientation of erythrocytes with respect to the applied forces. Deformability is an intrinsic property of erythrocytes that is determined by the geometric and material properties of this unique cell type [15].

Rheometer is the instrument used to measure a material's rheological properties. There are many types of rheometers that are available with the most versatile being controlled stress and/or strain rheometer and capillary rheometers [10].

The size of NPs is similar to that of most biological molecules; thus, an increased attention is focused on the applications of NPs in biology and medicine. Therefore, further additional rheological property studies should be performed [16].

The release of drug from semi-solid carriers is influenced by the rheological behaviour as well. The effect of certain parameters such as storage time, and temperature on the quality of GNPs as pharmaceutical products can be also investigated via rheological measurements.

Rheological alterations due to physical, dimensional and morphological changes of GNPs have not been previously documented. To understand and categorize the role of GNPs in drug delivery and cancer therapy, rheological information of GNPs should be evaluated through: 1) fixed temperature and varied shear rates; 2) varied temperature and fixed shear rates in the presence of GNPs of varying size, number of particles, shape and surface. Very little information on these aspects is presently available, which implies an urgent need for rheological data related to GNPs.

## Materials and methods

### GNPs size

10, 20 and 50 nm GNPs in aqueous solution (Product MKN-Au; M K Impex Corp, Canada) was used in this study. The GNPs were of size 10 nm (Product MKN-Au-010: concentration 0.01% Au; 5.7 × 10^12 ^particles/ml), of size 20 nm (Product MKN-Au-020: concentration 0.01% Au; 7.0 × 10^11 ^particles/ml), and of size 50 nm (Product MKN-Au-050: concentration 0.01% Au; 4.5 × 10^11 ^particles/ml).

### Experimental set up and rheological parameters measurement

The rheological parameters were viscosity (cp), torque%, shear stress (dyne/cm^2^) and shear rate (s^-1^). These rheological parameters were measured using Brookfield LVDV-III Programmable rheometer (cone-plate viscometer; Brookfield Engineering Laboratory, Incorporation, Middleboro, USA), supplied with temperature bath and controlled by a computer.

The rheometer was guaranteed to be accurate within ± 1% of the full scale range of the spindle/speed combination in use reproducibility is within ± .2%. Temperature inside the sample chamber was carefully monitored using a temperature sensor during the measurement of rheological parameters.

A cone and plate sensor having a diameter of 2.4 cm with an angle of 0.8 was used. The rheometer was calibrated using the standard fluids. This viscometer has a viscosity measurement range of 1.5-30,000 mPas and can handle the viscosity measurement results within the temperature range of this experiment.

The spindle type (SC-40) and its speed combinations will produce results with high accuracy when the applied torque is in the range of 10% to 100% and accordingly the spindle is chosen.

0.5 ml of each GNP size was poured in the sample chamber of the rheometer. The spindle was immersed and rotated in these gold nanofluids in the speed range from 50 to 250 RPM in steps of 20 minutes. The viscous drag of the GNP aqueous solution against the spindle was measured by the deflection of the calibrated spring.

## Results and discussion

### Dimension and morphology of GNPs

The mean size for GNPs was calculated from the images taken by the transmission electron microscope (TEM). The 10 nm GNPs excepted mean size of 9.45 ± 1.33 nm, 20.18 ± 1.80 nm for GNPs of size 20 nm and 50.73 ± 3.58 nm for GNPs of size 50 nm. Moreover, the 10 and 20 nm GNPs showed spherical morphology while 50 nm GNPs showed hexagonal morphology. The high electron densities of GNPs as well as the homogeneity of particles shape and size make them highly conspicuous under the TEM.

### Rheological parameters

**Figure 1:**
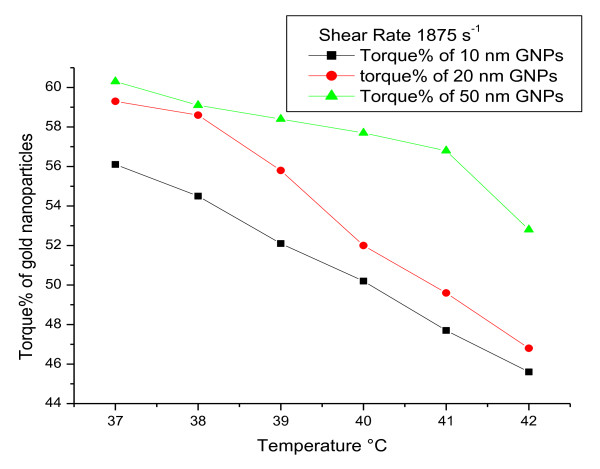
**The relation between torque% and temperature (°C) for 10, 20 and 50 nm GNPs at a fixed shear rate**.

**Figure 2:**
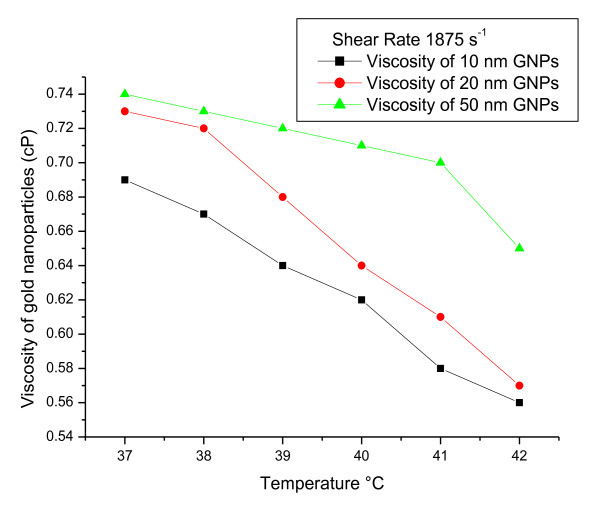
**The relation between viscosity (cp) and temperature (°C) for 10, 20 and 50 nm GNPs at a fixed shear rate**.

**Figure 3:**
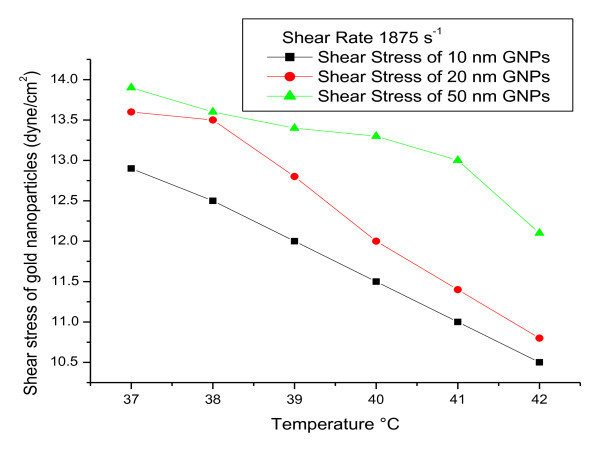
**The relation between shear stress and temperature°C for 10, 20 and 50 nm GNPs at a fixed shear rate**.

To measure viscoelasticity of GNPs (e.g., viscosity as a function of temperature), rheological parameters were measured at a gradual increase of temperature from 37 to 42°C and fixed shear rate of 1875 s^-1 ^**(**Figures 1, 2 and 3**)**. The torque% **(**Figure 1**)**, viscosity **(cp) (**Figure 2**) **and SS (dyne/cm^2^) **(**Figure 3**) **of all GNP sizes decreased with increasing the temperature and with decreasing the GNP size (for each fixed temperature value).

**Figure 4:**
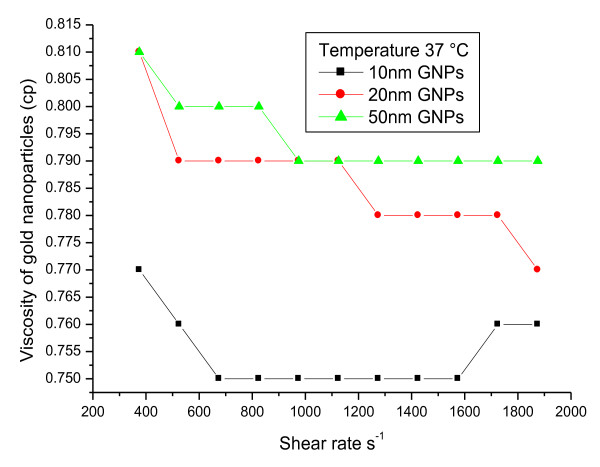
**The relation between viscosity and shear rate for 10, 20 and 50 nm GNPs at a fixed temperature**.

To measure fluid properties of GNPs (viscosity as a function of shear rate) to determine whether the flow is Newtonian or non-Newtonian behaviour, rheological parameters were measured at starting temperature of 37°C and wide range of shear rates from 375 to 1875 s^-1^. The relation between viscosity (cp) and shear rate (s^-1^) for 10, 20 and 50 nm GNPs at a temperature of 37°C showed non-Newtonian behaviour **(**Figure 4**)**. For each shear rate value, the viscosity of all GNPs decreased with decreasing the GNP size.

**Figure 5:**
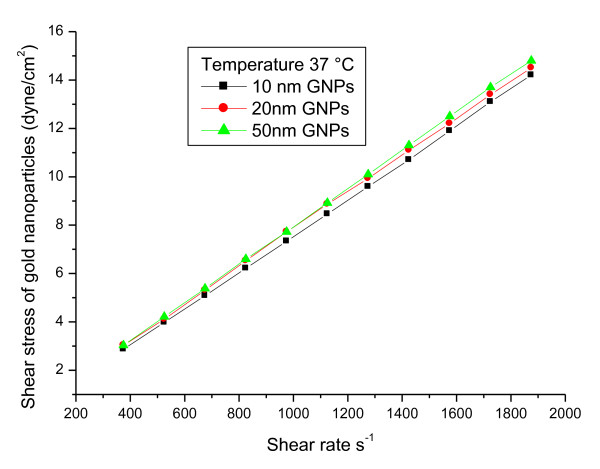
**The relation between shear stress and shear rate for 10, 20 and 50 nm GNPs at a fixed temperature**.

Although the relationship between SS (dyne/cm^2^) and SR (s^-1^) for 10, 20 and 50 nm GNPs was linearly related however the fluid properties of GNPs showed non-Newtonian behaviour as shown in Figure 5. The fitted linear relationship between SS and SR for GNPs can be given by:

**10 nm GNPs: **   SS = 1.006 SR - 4.956   R^2 ^= 0.9999

**20 nm GNPs: **   SS = 0.995 SR - 4.899   R^2 ^= 0.9999

**50 nm GNPs: **   SS = 1.001 SR - 4.915   R^2 ^= 1

Abdelhalim and Jarrar have reported that fatty change was observed in some swelling hepatocytes of rats exposed to 100 μl of 10 nm GNPs and to lesser extent in the ones exposed to larger particles. This hepatic liposis was more prominent in rat exposed to GNPs for 7 days than those received the treatment for 3 days. Hepatocytes fatty change might be due to lipid peroxidation that leads to rough endoplasmic damage and detachment of the cytoplasmic lipoprotein which indicate abnormal fat metabolism [17-20]. Nitric oxide (NO) reacts rapidly with superoxide producing peroxynitrite (ONOO-) which can interact with lipids, DNA, and proteins via direct oxidative reactions or via indirect radical-mediated damage. ROS production could result from the proportionately high surface area of GNPs used in this investigation [21-23].

It has been reported that 5 nm GNPs caused significantly greater oxidative stress and cytotoxicity effects than larger ones [24]. The 5 nm GNPs have shown to catalyze NO production from endogenous S-nitroso adducts with thiol groups in blood serum.

The hepatocytes have shown abnormal retention of lipids induced by GNPs which might indicate toxic injury to liver in the form of hepatocytes liposis by these particles [25].

To evaluate the effects of cholesterol, triglycerides and other lipid constituents on the rheological properties of GNPs, further experimental work using animals fed high cholesterol diet and injected with GNPs for long period of time at least for 12 weeks or more is now performed.

## Conclusions

The present study attempted to characterize the rheological properties of different sizes of GNPs at: 1) fixed temperature and wide range of shear rates; 2) varied temperature and fixed shear rate.

Several rheological parameters of GNPs such as viscosity (cp), torque%, shear stress (dyne/cm^2^) and shear rate (s^-1^) were evaluated using Brookfield LVDV-III Programmable rheometer supplied with temperature bath and controlled by a computer.

The 10, 20 and 50 nm GNPs showed mean size of 9.45 ± 1.33 nm, 20.18 ± 1.80 nm, and 50 nm GNPs, respectively. The 10 and 20 nm GNPs showed spherical morphology while the 50 nm GNPs showed hexagonal morphology using the transmission electron microscope (TEM).

The relation between viscosity (cp) and shear rate (s^-1^) for 10, 20 and 50 nm GNPs at a temperature of 37°C showed non-Newtonian behaviour. The relationship between SS (dyne/cm^2^) and SR (s^-1^) for 10, 20 and 50 nm GNPs showed non-Newtonian behaviour.

The torque%, viscosity **(cp) **and SS (dyne/cm^2^) of all GNP sizes decreased with increasing the temperature and with decreasing the GNP size (for each fixed temperature value).

For each SR (s^-1^) value, the viscosity of all GNPs decreased with decreasing the GNP size.

This study demonstrates that the physical, dimensional and morphological changes of GNPs have effective influence on their rheological properties.

To understand and categorize the role of GNPs in drug delivery and cancer therapy, GNPs of varying size, number of particles, shape and surface should be taken into consideration. Moreover, further additional in vivo and in vitro studies after administration of GNPs should be performed to support this hypothesis.

## Competing interests

The author declares that he has no competing interest.

## Authors' contributions

AMAK analysed, interpreted the data and wrote the final draft of this manuscript. The animal model used in this study was obtained from the Laboratory Animal Centre (College of Pharmacy, King Saud University, Saudi Arabia). AMAK conceived the study and its design and obtained research grants for this study. The author has read and approved the final manuscript.
